# Dietary Phytochemicals in Neuroimmunoaging: A New Therapeutic Possibility for Humans?

**DOI:** 10.3389/fphar.2016.00364

**Published:** 2016-10-13

**Authors:** Graziamaria Corbi, Valeria Conti, Sergio Davinelli, Giovanni Scapagnini, Amelia Filippelli, Nicola Ferrara

**Affiliations:** ^1^Department of Medicine and Health Sciences, University of MoliseCampobasso, Italy; ^2^Department of Medicine, Surgery and Dentistry, University of SalernoSalerno, Italy; ^3^Department of Translational Medical Sciences, Federico II University of NaplesNaples, Italy; ^4^Salvatore Maugeri Foundation, IRCCS, Scientific Institute of TeleseTelese Terme, Italy

**Keywords:** antioxidants, Nfr2, resveratrol, sirtuins, curcumin

## Abstract

Although several efforts have been made in the search for genetic and epigenetic patterns linked to diseases, a comprehensive explanation of the mechanisms underlying pathological phenotypic plasticity is still far from being clarified. Oxidative stress and inflammation are two of the major triggers of the epigenetic alterations occurring in chronic pathologies, such as neurodegenerative diseases. In fact, over the last decade, remarkable progress has been made to realize that chronic, low-grade inflammation is one of the major risk factor underlying brain aging. Accumulated data strongly suggest that phytochemicals from fruits, vegetables, herbs, and spices may exert relevant immunomodulatory and/or anti-inflammatory activities in the context of brain aging. Starting by the evidence that a common denominator of aging and chronic degenerative diseases is represented by inflammation, and that several dietary phytochemicals are able to potentially interfere with and regulate the normal function of cells, in particular neuronal components, aim of this review is to summarize recent studies on neuroinflammaging processes and proofs indicating that specific phytochemicals may act as positive modulators of neuroinflammatory events. In addition, critical pathways involved in mediating phytochemicals effects on neuroinflammaging were discussed, exploring the real impact of these compounds in preserving brain health before the onset of symptoms leading to inflammatory neurodegeneration and cognitive decline.

## Introduction

In the last decades the increasing aging with consequent raise in chronic degenerative diseases has led to an augmented investigation of the environmental factors involved in their origin and progression.

Although several efforts have been made in the search for genetic and epigenetic patterns linked to diseases, a comprehensive explanation of the mechanisms underlying pathological phenotypic plasticity is still far from being clarified (Babenko et al., 2012). Epigenetic control of the gene expression has been recognized as a key player in producing rapid adaptation to changing environmental conditions both within a single lifespan as well as across multiple generations. These mechanisms are particularly applied to the brain, which is capable of changing readily in response to experience throughout a lifetime (Babenko et al., 2012).

Epigenetics is a branch of biology, which studies how changes in gene expression occur without modifications in the DNA sequence (Choudhuri, 2011). Such changes can be induced by environmental factors, and can be highly stable, including those resulting from genetic imprinting, or dynamic, including those associated with memory (Lardenoije et al., 2015). Oxidative stress and inflammation are two of the major triggers of the epigenetic alterations occurring in chronic pathologies, such as neurodegenerative diseases, especially in elderly population, already characterized by modifications of the normal homeostasis of organs and systems.

Starting by the evidence that a common denominator of aging and chronic degenerative diseases is represented by inflammation, and that several dietary phytochemicals are able to potentially interfere with and regulate the normal function of cells (Scapagnini et al., 2014), in particular neuronal components, aim of this review is to summarize recent studies on the neuroinflammaging processes indicating that specific phytochemicals may act as positive modulators of neuroinflammatory events. In addition, pathways involved in mediating phytochemicals effects on neuroinflammaging were discussed, exploring the real impact of dietary phytochemicals in preserving brain health before the onset of symptoms leading to inflammatory neurodegeneration and cognitive decline.

## Features of neuroimmunoaging

Over the last decade, remarkable progress has been made to realize that chronic, low-grade inflammation is one of the major risk factor underlying brain aging. During their life the cells progressively impair the ability to defend themselves from stress stimuli and, as a consequence, there is an accumulation of oxidative damages in all cell constituents (Corbi et al., 2008; Bianco et al., 2013; Lardenoije et al., 2015; Conti et al., 2015, 2016).

Growing evidence suggests that the brain and immune system are intricately connected and crosstalk to maintain homeostasis.

**Figure 1:**
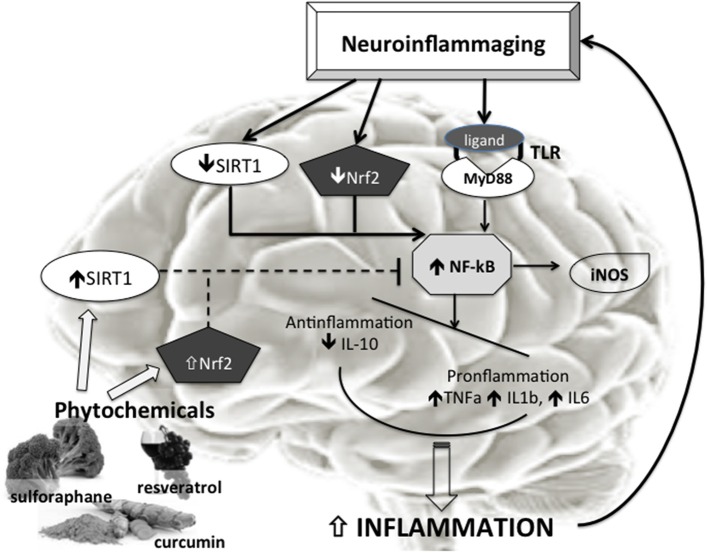
**Phytochemicals effects on Neurooinflammaging**. Neuroimmunoinflammaging is characterized by reduced SIRT1 and Nfr2 activity with consequent increased NF-κB activation. The increased NF-κB activation, also trough Tool Like Receptors (TLR), induces in turn raised proinflammatory factors such as TNFa, IL1b, IL6, iNOS. The disequilibrium between anti- (IL10) and pro-inflammatory molecules determines increased inflammation, and a vicious circle is established that sustains neuroinflammaging. The phytochemicals (like curcumin, resveratrol, sulphurane, etc.) inducing increase in Nrf2 and SIRT1 activity could be able to inhibit the NF-κB activation and then to break the vicious circle ending the progression of the brain aging.

Aging is associated with aberrant inflammatory responses in human brains (Lu et al., 2004; Cribbs et al., 2012). Specifically, basal levels of proinflammatory cytokines are elevated with aging (Sierra et al., 2007), whereas anti-inflammatory mediators are reduced (Ye and Johnson, 1999). In addition, other components involved in innate immune responses, such as the complement (C) pathway, the toll-like receptor (TLR) signaling, and the inflammasome activation, are also upregulated as the brain ages (Cribbs et al., 2012; Cho et al., 2015). In fact, during aging the brain shows an imbalance between pro-and anti-inflammatory cytokine levels (Figure 1).

It has been demonstrated both in humans and animal models that aging is associated with decreased levels of interleukin 10 (IL10) (Ye and Johnson, 2001), and increased levels of tumor necrosis factor alpha (TNFa) and IL1b in the nervous system (Lukiw, 2004; Streit et al., 2004), as well as IL6 in plasma (Ye and Johnson, 2001; Godbout and Johnson, 2004). In addition, increased levels odtransforming growth factor b1 (TGFb1) mRNA, a key regulatory cytokine, has been observed in the brain of aged mice and rats (Bye et al., 2001). At the same time, several changes induced by an aged micro-environment, such as increased systemic inflammation, increased permeability of the blood-brain barrier (BBB), and degeneration of neurons and other brain cells, could contribute to the production of Radical Oxygen Species (ROS), thus generating oxidative stress. It has been proposed that BBB permeability increases in aged animals (Blau et al., 2012; Enciu et al., 2013), facilitating perhaps infiltration by monocytes releasing mitochondria-generated ROS. According to this hypothesis, an age-related increase in the number of CD11bC and CD45 cells, compatible with infiltrated monocytes, has been reported in the brain of aged rats (Blau et al., 2012). Likewise, expression levels of chemotactic molecules, such as interferon-inducible protein 10 (IIP10) and monocyte chemotactic protein-1 (MCP-1), are increased in the hippocampal region (Blau et al., 2012; Von Bernhardi et al., 2015).

With normal aging, the immunophenotype of microglia is characterized by up-regulation of glial activation markers including Major Histocompatibility Complex II (MHC II) and CD11b, a finding reported in several species including human post-mortem tissue, rodent, canine, and non-human primates (Tafti et al., 1996; Sheffield and Berman, 1998). This up-regulation of MHCII occurs also at the mRNA level (Frank et al., 2006). Importantly, MHCII is expressed at very low levels on microglia of younger animals under basal conditions (Perry, 1998), providing a clear baseline to detect aging-related changes in microglia immunophenotype. Increased MHCII could result from aging-induced increases in microglia number, or from increases in permicroglial cell expression. Although only few studies are available, they support the idea of increased permicroglial cell expression, and therefore sensitization (Barrientos et al., 2015). Despite these commonalities, the role of the immune system in aging and neurodegenerative disease remains unclear (Lucin and Wyss-Coray, 2009).

Microglia are the resident immune cells of the brain, endowed with numerous receptors capable of detecting physiological disturbances. When neurons are injured as a result of aging or neurodegeneration, microglia become activated via the release of adenosine triphosphate (ATP), neurotransmitters, growth factors or cytokines, ion changes in the local environment, or loss of inhibitor molecules displayed by healthy neurons (Hanisch and Kettenmann, 2007).

The increase in expression of multiple TLRs in the aging brain (Letiembre et al., 2007; Berchtold et al., 2008) may generate a hypersensitive state of glia and neurons and thus magnify potential injury. Stimulation of TLRs induces a signaling cascade, culminating in the activation of nuclear factor κB (NFκB) and subsequent transcriptional activation of numerous proinflammatory genes, encoding cytokines, chemokines, complement proteins, enzymes [such as cyclooxygenase 2 (COX-2) and Inducible nitric oxide synthase (iNOS)], adhesion molecules, and immune receptors (Nguyen et al., 2004). Exactly how neuronal TLRs promote neurodegeneration and the identity of their ligands is currently unclear.

However, aging results in a significant increase in glial activation, complement factors, inflammatory mediators, and brain atrophy (West et al., 1994; Streit et al., 2008). Microarrays of aged human and mouse brains showed that genes related to cellular stress and inflammation increase with age while genes related to synaptic function/transport, growth factors, and trophic support decrease (Lee et al., 2000). These changes suggest that neurons encounter increased challenges with age but receive reduced support. Neurogenesis also decreases with age, possibly as a result of factors secreted by activated microglia (i.e., IL-6). To date, it is unclear the reason of increased inflammation during aging. However, genetic studies suggest an important role of Deoxyribonucleic Acid (DNA), because DNA bases are particularly vulnerable to oxidative stress damage leading to important inflammatory alterations (Bianco et al., 2013). Also unclear is to what extent aging affects the responsiveness of microglia or their potential to contribute to neuronal loss. Despite morphological and phenotypic changes that indicate microglial activation, it has been proposed that microglia may actually become dysfunctional and enter a senescent state with age (Streit et al., 2008). Such a state may cause microglia to secrete diminished levels of neurotrophic factors and downregulate phagocytic function. This phenomenon, associated with increased secretion of inflammatory mediators, may lead to neuronal loss and inefficient clearance of toxic protein aggregates in neurodegenerative disease (Lucin and Wyss-Coray, 2009). Finally, it should be also highlighted that mounting evidence indicates that epigenetic mechanisms play a significant role in shaping environmental influences on brain and behavior (Kosik et al., 2012).

## Nrf2 and sirtuins pathway involved in neuroimmunoaging

Although definitive mechanisms are still to be elucidated, the pro-inflammatory phenotype of senescent cells, coupled with the up-regulation of the inflammatory response with increasing age, has been found to play a role in the initiation and progression of age-related diseases such as Alzheimer's disease (Cevenini et al., 2013; Patel et al., 2015).

A large body of evidence has highlighted a role of class III histone deacetylases, named sirtuins, in neurodegenerative processes (Kim et al., 2007; Vang et al., 2011; Baur et al., 2012). Sirtuin 1 (SIRT1), the main characterized molecule of sirtuins family, regulates immune responses via NF-κB signaling and in this way also controls the ROS production (Salminen et al., 2013).

The NF-κB signaling is a crucial pathway of immune defense system and an inducer of inflammatory responses (Vallabhapurapu and Karin, 2009). The NF-κB system is involved in many housekeeping and survival functions during cellular stress e.g., by controlling apoptosis, proliferation, and energy metabolism (Karin and Lin, 2002; Perkins, 2007; Johnson and Perkins, 2012). Both SIRT1 and oxidative stress are known to be able to regulate NF-κB signaling and are crucially involved in the maintenance of cellular homeostasis (Yeung et al., 2004; Morgan and Liu, 2011). Moreover, several studies demonstrated that NF-κB signaling is activated during aging (Helenius et al., 2001; Csiszar et al., 2008).

The crosstalk between oxidative stress and inflammation is a complex process and there are studies reporting that ROS can stimulate inflammation via the activation of inflammasomes and the production of IL-1β and IL-18 cytokines, which subsequently trigger inflammatory responses (Kitazawa et al., 2005; Heneka and O'Banion, 2007; Salminen et al., 2013). Many studies have also demonstrated that SIRT1 is a potent intracellular inhibitor of oxidative stress and inflammatory responses (Rajendran et al., 2011; Salminen et al., 2011). In particular, SIRT1 is a powerful inhibitor of NF-κB signaling and thus it suppresses inflammation (Yeung et al., 2004; Salminen et al., 2008a). Many downstream targets of SIRT1 also repress inflammatory responses, e.g., AMP-activated protein kinase (AMPK) (Salminen et al., 2011) and Forkhead box O (FoxO) factors (Lin et al., 2004), by inhibiting the NF-κB signaling. Yeung et al. (2004) revealed that SIRT1 performs its antinflammatory activity by deacetylating the Lys310 residue of v-rel avian reticuloendotheliosis viral oncogene homolog A/p65 (RelA/p65) component, thus inhibiting the transactivation capacity of the NF-κB complex. Recently Cho et al. (2015) showed that SIRT1 levels in microglia exhibit an age-dependent decline, and microglial SIRT1 deficiency leads to cognitive decline in normal aging. The authors suggested that aging-induced SIRT1 deficiency in microglia could initiate epigenetic alterations on IL-1beta, leading to its enhanced expression that is associated with impairments in memory and related cognitive decline.

Moreover, there is a growing body of evidence suggesting that activation of SIRT1 and other sirtuins can protect neurons in experimental models of neurodegenerative disorders (Duan, 2013). In particular Min et al. (2010) demonstrated that tau protein, that stabilizes microtubules, is acetylated and tau acetylation prevents degradation of phosphorylated tau (p-tau). Hyperphosphorylation of the tau protein can result in the self-assembly of tangles of paired helical filaments and straight filaments, which are involved in the pathogenesis of Alzheimer's disease, and other tauopathies (Alonso et al., 2001). Deleting SIRT1 enhanced levels of acetylated-tau and pathogenic forms of p-tau, probably by blocking proteasome-mediated degradation. These results indicate that SIRT1 can prevent the formation of neurofibrillary tangles (Min et al., 2010; Lee et al., 2014). In primary cortical cultures, overexpression of SIRT1 in microglia protected against amyloid beta toxicity, most likely by inhibiting NF-κB signaling (Chen et al., 2005). SIRT1 could also protect against cellular senescence by inactivating NF-κB (Rovillain et al., 2011; Tilstra et al., 2012) or deacetylating the FOXO3 transcription factor (Yao et al., 2012). In addition, SIRT1 could also enhance the T helper 2 (Th2) lymphocytes responses in dendritic cells (Legutko et al., 2011).

In the Wallerian degeneration slow (Wlds) mouse model, SIRT1 activation protects axons against neuronal injury (Dali-Youcef et al., 2007). Decreasing SIRT1 activity reduces the axonal protection originally observed, whereas SIRT1 activation by resveratrol decreases the axonal degeneration after neuronal injury (Suzuki and Koike, 2007). This suggests that the neuroprotection in the Wild mouse model is achieved by increasing the neuronal nicotinamide adenine dinucleotide (NAD+) reserve and/or SIRT1 activity (Dali-Youcef et al., 2007). Also the inhibition of sirtuin 2 (SIRT2) rescued a-synuclein toxicity and modified inclusion morphology in a cellular model of Parkinson's disease and genetic inhibition of SIRT2 via small interfering RNA similarly rescued a-synuclein toxicity (Outeiro et al., 2007). Furthermore, the inhibitors protected against dopaminergic cell death both *in vitro* and in a Drosophila model of Parkinson's disease, suggesting another link between neurodegeneration and aging (Outeiro et al., 2007). In addition, SIRT1 activation significantly decreases neuronal cell death induced by amyloid-beta peptides through inhibition of NF-κB signaling (Dali-Youcef et al., 2007). In particular SIRT1 deacetylates retinoic acid receptor beta (RARb) and activates a disintegrin and metalloprotease domain (ADAM) 10 transcription, leading to upregulated Amyloid Precursor Protein (APP) processing by a-secretase, resulting in reduced production of Amyloid beta (Aβ) peptide (Donmez et al., 2010). Thanks to these evidences, SIRT1, as well as the other sirtuins, is now considered a promising therapeutic option for neurological syndromes, such as Alzheimer, Parkinson, and Huntington's disease (Donmez et al., 2010; Jeong et al., 2012), and in general for the control and progression of the neuroimmunoaging.

Another key molecule involved in neuroimmunoaging is represented by Nuclear factor (erythroid-derived 2)-like 2 (Nrf2). Emerging evidence suggests that Nrf2 may play an important role in the regulation of brain inflammation, and some studies have suggested that Nrf2 has an antagonistic effect with the NF-κB pathway, which is considered a hallmark of inflammation (Liu et al., 2008; Djordjevic et al., 2015). Nrf2 is a member of the Cap‘n’Collar family of transcription factors that bind to nuclear factor erythroid derived 2 (NF-E2) binding sites (GCTGAGTCA) that are essential for the regulation of erythroid specific genes. Nrf2 is expressed in a wide range of tissues, many of which are sites of expression for phase 2 detoxification genes (Dinkova-Kostova et al., 2002) and targeted for ubiquitination and proteasomal degradation via binding to a cytosolic repressor protein, Kelch-like ECH associated protein 1 (Keap1) (McMahon et al., 2006). The principle of the Nrf2 system is to keep Nrf2 protein low under normal conditions with the possibility of rapid induction in case of a sudden increase in oxidation status in the cell. This is achieved by constitutive synthesis and degradation of Nrf2 with the possibility of rapid redirection of Nrf2 to the nucleus. (Sandberg et al., 2014). There is now overwhelming amount of experimental evidence that Nrf2 serves as a master regulator of the antioxidants involved in cellular defenses against various electrophiles and oxidants (Kobayashi and Yamamoto, 2006; Calabrese et al., 2008). Indeed new findings connect Nrf2 also to expression of other types of protective proteins such as brain derived neurotrophic factor (BDNF) (Sakata et al., 2012), the anti-apoptotic B-cell lymphoma 2 (BCL-2) (Niture and Jaiswal, 2012), the anti-inflammatory interleukin (IL)-10, the mitochondrial transcription (co)-factors NRF-1 and peroxisome proliferator-activated receptor gamma coactivator 1-alpha (PGC-1α) (Piantadosi et al., 2011).

The relation between Nrf2 and NF-κB is not well characterized but the identification of NF-κB binding sites in the promoter region of the Nrf2 gene suggests cross talk between these two regulators of inflammatory processes (Nair et al., 2008). The NF-κB subunit p65 has been shown to function as a negative regulator of Nrf2 activation either by depriving cAMP response element-binding protein (CBP) from NRF2 or by recruitment of histone deacetylase 3 (HDAC3), causing local histone hypoacetylation and down-regulation of Nrf2-antioxidant responsive element (Nrf2-ARE) signaling (Liu et al., 2008). Yu et al. (2011) have shown that p65 decreased Nrf2 binding to its cognate DNA sequences and enhanced Nrf2 ubiquitination, then providing direct evidence that the interaction of nuclear factor p65 with Keap1 is critical for NF-κB repressing Nrf2-ARE pathway. Moreover, the N-terminal region of p65 was necessary for both the interaction with Keap1 and its transcriptional suppression activity, and nuclear translocation of Keap1 was augmented by p65. The authors concluded that taken together, these findings suggest that NF-κB signaling inhibits Nrf2-ARE pathway through the interaction of p65 and Keap1 (Yu et al., 2011). Further, activation of NRF2 in response to lipopolysaccharide (LPS) has been suggested to be dependent on the key innate immunity-regulating adaptor protein Myeloid differentiation primary response gene 88 (MyD88) (Kim et al., 2011). In particular Kim et al. (2011) demonstrated that treatment of macrophages with LPS activates Nrf2. Interestingly, the authors found that Nrf2 is activated in a MyD88 dependent fashion without the involvement of ROS. These results suggest the possibility that Nrf2 activated by inflammatory stimuli can be a mechanism that contributes to decreasing excessive inflammatory response (Kim et al., 2011). A study demonstrated that Nrf2 knockout mice were hypersensitive to the neuroinflammation induced by LPS, indicative of an increase in microglial cells, and in the inflammation markers iNOS, IL-6, and TNF-α, compared with the hippocampi of wild-type littermates (Innamorato et al., 2008). Activation of NRF2 could also be achieved via the increase in peripheral IGF-1 that enters the brain after exercise (Cotman et al., 2007; Sandberg et al., 2014).

Recently Rojo et al. (2010) showed that Nrf2-deficient mice exhibited more astrogliosis and microgliosis. Inflammation markers characteristic of classical microglial activation, COX-2, iNOS, IL-6, and TNF-α were also increased and, at the same time, anti-inflammatory markers attributable to alternative microglial activation, such as FIZZ-1 and IL-4 were decreased. These results were confirmed in microglial cultures, further demonstrating a role of Nrf2 in tuning balance between classical and alternative microglial activation (Rojo et al., 2010).

Aging drives a long-lasting sub-ventricular zone impairment at least in part via reduced Nrf2-mediated tolerance to inflammation and oxidative stress associated with dysfunctional astrocyte–microglial dialogue, in turn interrupting key molecular signaling mechanisms finely regulating sub-ventricular cell homeostasis (L'Episcopo et al., 2013). In particular, when “primed” microglia of aged mice become hyperactivated upon a second hit, the generation of highly toxic mediators in the face of impaired antioxidant self-protective neuroprogenitor cell response dramatically inhibits neurogenesis, suggesting that glial age is of critical importance in directing promotion vs. inhibition of neurogenesis. Interestingly, with age, the exaggerated microglial activation can impair an astrocyte's ability to express critical antioxidant, anti-inflammatory, and neurogenic factors, thereby resulting in an overall reduction of glial proneurogenic capacities (L'Episcopo et al., 2013). These processes may disrupt the cross talk between two pivotal pathways in sub-ventricular zone, the NrF2/ Fosfoinositide 3-chinasi/Protein kinase B (Nrf2/PI3-K/Akt) and the *Drosophila melanogaster* wingless gene/receptor Frizzled/β-catenin (Wnt/Fzd/β-catenin) signaling cascades, involved in cell survival, proliferation, and/or differentiation. The manipulation of these age-related Nrf2 pathways at middle age is associated with significant Dopaminergic neuroprotection (L'Episcopo et al., 2013). A study also demonstrated that direct intrahippocampal gene delivery of Nrf2, by a lentiviral vector, results in a reduction in spatial learning deficits in aged mice (Kanninen et al., 2009). In particular memory improvement in the mice after Nrf2 transduction shifts the balance between soluble and insoluble Aβ toward an insoluble Aβ pool without concomitant change in total brain Aβ burden. Nrf2 gene transfer was associated with reduction in astrocytic but not microglial activation and induction of Nrf2 target gene Heme Oxygenase 1 (HO-1), indicating overall activation of the Nrf2-ARE pathway in hippocampal neurons 6 months after injection (Kanninen et al., 2009). Based on this body of emerging evidence it seems that in many cases the beneficial effects of low doses of phytochemicals rely on their ability to activate the Nrf2/ARE and sirtuins pathways.

## Mechanisms of dietary phytochemicals in neuroimmunoaging

Dietary phytochemicals include a large group of no-nutrients compounds from a wide range of plant-derived foods and chemical classes. Several plant-based extracts and chemicals are supposed to have beneficial effects on human brain function. The potential effect of these molecules is linked to the common ancestry, which has provided some phytochemicals of conserved cellular processes, including the similarities in most pathways for synthesis and breakdown of proteins, nucleic acids, carbohydrates, and lipids (Kennedy and Wightman, 2011). In fact, some molecules that function as neurochemicals within the mammalian central nervous system (CNS) are ubiquitous across all eukaryotes (Kawashima et al., 2007).

At a molecular level, signaling molecules and pathways are preserved in both plants and animals (Kushiro et al., 2003). For instance, multiple aspects of cellular and redox signaling are conserved (Dalle-Donne et al., 2009), including similar gene expression in response to cellular stressors, which are regulated by common transcription factors (Scandalios, 2005).

The basis for the use of polyphenol-rich nutritional supplements as a modulator of age-related cognitive decline is the age-related increase in oxidative stress (Morris et al., 2006; Craft et al., 2012) and low-grade inflammation.

Often the beneficial effects of phytochemicals are supposed to be due to their intrinsic antioxidant and antinflammatory properties (Murugaiyah and Mattson, 2015).

At low doses, phytochemicals have beneficial or stimulatory effects on animal cells, whereas in high amounts can be toxic. This is an example of “hormesis” (Mattson, 2008; Lee et al., 2014). Hormetic phytochemicals such as resveratrol, sulforaphane, curcumin, catechins, allicin, and hypericin are reported to activate adaptive stress response signaling pathways that increase cellular resistance to injury and disease (Mattson and Cheng, 2006).

Also neuroactive phytochemicals present in commonly consumed fruits, vegetables, and nuts are generally well tolerated (Wöll et al., 2013; Lee et al., 2014). These phytochemicals are hormetic substances because they can be toxic in high amounts, but are beneficial in the lower amounts usually consumed (Mattson, 2015). In this context, although controversial data are available on the capacity of these compounds to cross the BBB and bioavailability continues to be highlighted as a major concern, hormetic dose-response model has important biological and clinical implications, including activation of neuroprotective stress response pathways at low concentrations (Schaffer and Halliwell, 2012; Davinelli et al., 2016).

Their effects are represented as a biphasic dose–response curve, with the first phase being a positive/beneficial effect and the second phase with a progressively negative/toxic effect (Calabrese et al., 2007; Mattson et al., 2007).

Recent findings suggest that adaptive cellular stress responses to phytochemicals are mediated via some of the same pathways that mediate responses to energy restriction and exercise (Mattson, 2012; Milisav et al., 2012). Commonly consumed phytochemicals are able to induce mild stress in neural cells, enhancing the ability of nervous system to cope with stress, and then promoting optimal function and longevity of the nervous system. As with exercise and energy restriction, intake of neurohormetic phytochemicals typically occurs on intermittent basis, which provides a “recovery period” that allows cells to repair and growth (Mattson, 2015). Examples include pathways that signal via Nrf2, SIRT1, and AMPK (Menendez et al., 2013; Misra et al., 2013). Activation of one or more of these signaling pathways that evolved to defend cells against potentially toxic phytochemicals appears to be a major reason why ingestion of these substances can protect neurons against injury and disease (Calabrese et al., 2008; Murugaiyah and Mattson, 2015).

## Some phytochemicals potentially useful in neuroprotection

Several studies indicate that antioxidants, e.g., dietary polyphenols, can inhibit inflammation, and in particular the terpenoids, are able to inhibit NF-κB signaling and thus repress inflammation (Rahman et al., 2006; Salminen et al., 2008b).

In this context, there are many mutual interactions and a delicate balance exists between SIRT1 and ROS signaling which provoke context-dependent responses to autophagic flux and inflammation (Salminen et al., 2013).

Phytochemicals like curcumin, resveratrol, terpenoids, epigallocatechin-3-gallate (EGCG), and isothiocyanates share common properties and play an important role to activate the phase II detoxifying and antioxidant enzymes like HO-1, glutathione peroxidase (GSH-Px), and glutathione-S-transferase (GST) by targeting the common transcription factor Nrf2 (Liby et al., 2007; Tosetti et al., 2009).

Recent findings suggest that several phytochemicals exhibit biphasic dose responses on cells with low doses activating signaling pathways that result in increased expression of genes encoding survival proteins, as in the case of the Keap1/Nrf2/ARE pathway activated by curcumin and NAD/NADH-sirtuin-1 activated by resveratrol.

To underline the role of the dietetic components in modifying cellular mechanisms, recently Morrison et al. (2010) showed as in 20-month old male mice fed either ‘western diet’ (41% fat), very high fat lard diet (60% fat), or corresponding control diets for 16 weeks, only the high fat lard diet increased age-related oxidative damage and impaired retention in the behavioral test. This selective increase in oxidative damage and cognitive decline was also associated with a decline in Nrf2 levels and activity, suggesting a potential role for decreased antioxidant response. Then the authors suggested that impaired Nrf2 signaling and increased cerebral oxidative stress as mechanisms underlying High Fat Diet-induced declines in cognitive performance in the aged brain (Morrison et al., 2010).

### Ferulic acid

Ferulic acid (FA) is commonly found in fruits and vegetables such as tomatoes, sweet corn, and rice (Srinivasan et al., 2007). It has been reported that this compound decreases the levels of inflammatory mediators (prostaglandin E2 and TNF-α) (Ou et al., 2003), and iNOS expression and function (Tetsuka et al., 1996). *In vivo*, long-term administration of FA effectively protects against Aβ toxicity by inhibiting microglial activation (Kim et al., 2004).

Kanaski et al. (2002) reported that FA protects against free radical mediated changes in the conformation of synaptosomal membrane proteins. The long-term administration of FA at a dose of 300 μM effectively protects against Aβ toxicity by inhibiting microglial activation *in vivo* (Kim et al., 2004). Moreover, Sultana et al. (2005) showed that also at lower doses of 10–50 μM significantly protects against Aβ toxicity by modulating oxidative stress directly and by inducing protecting genes in hippocampal cultures, also exerting neuroprotective effects by up-regulation of protective enzymes, such as Hemeoxygenase-1 (HO-1) and heat shock protein 70 (Hsp70) (Scapagnini et al., 2004; Srinivasan et al., 2007) then suggesting a control by Nrf2 and sirtuins on the FA effects. More recently Mori et al. (2013) demonstrated that also orally administration of FA for 6 months improved behavioral impairment, mitigated cerebral amyloidosis, and inhibited APP metabolism by reducing β-site APP cleaving enzyme 1 (BACE1) expression and β-secretase activity in an accelerated mouse model of cerebral amyloidosis. Supporting results from cultured mutant human APP-overexpressing murine neuron-like cells revealed FA dose-dependent reduction of various Aβ species and inhibition of β-secretase cleavage. FA also ameliorated neuroinflammation in including β-amyloid plaque-associated gliosis and expression of the proinflammatory cytokines, TNF-α, and IL-1β. Lastly, mRNA expression of three oxidative stress markers [superoxide dismutase 1 (SOD1), catalase (CAT), and GSH-Px 1] was decreased in FA-treated mice, providing support for long-term FA dietary supplementation as a therapeutic strategy (Mori et al., 2013).

### Green tea

The consumption of green tea has recently attracted much attention in the occidental culture because of its beneficial effects, such as protection of dopaminergic neurons from damage induced by 6-hydroxydopamine in a rat model of Parkinson's disease (Guo et al., 2007); reduction of mutant huntingtin misfolding and neurotoxicity in Huntington's disease models (Ehrnhoefer et al., 2006); direct protection of neurons against Aβ toxicity (Bastianetto et al., 2000); protection against Aβ-induced cognitive impairment in a rat model relevant to Alzheimer's disease (Haque et al., 2008). Moreover, a study by Wu et al. (2006) reports that one of its component, epigallocatechin gallate (EGCG), up-regulates HO-1 expression by activation of the Nrf2-ARE pathway in endothelial cell, conferring resistance against Hydrogen peroxide (H2O2) induced cell death, suggesting a hormetic mechanism of action (Wu et al., 2006).

It has been demonstrated that EGCG selectively protects cultured rat cerebellar granule neurons from oxidative stress (Schroeder et al., 2009).

More recently Obregon et al. have confirmed that oral administration of EGCG promotes cleavage of APP into α-CTF and soluble APP-α (Obregon et al., 2006). These cleavage events are associated with elevated α-secretase cleavage activity and are also positively correlated with activation of ADAM10, a key candidate α-secretase (Rezai-Zadeh et al., 2008), then suggesting a role of sirtuins in mediating EGCG effects (Donmez et al., 2010; Jeong et al., 2012).

### Blueberry and strawberry

Evidences showed that plant extracts, from mulberry, strawberry, and blueberry, contain antioxidants, which are able to induce the antioxidant defense system and improve memory deterioration in aging animals (Shih et al., 2010).

Supplementation of the diet of 19 month-old rats with strawberry, blueberry or spinach extracts for 8 weeks resulted in the reversal of age-related deficits in several neuronal and behavioral parameters (Joseph et al., 1999). Blueberry supplementation prevented learning and memory deficits in a mouse model of Alzheimer's disease (Joseph et al., 2003). In addition, dietary supplementation with blueberry extract increased the survival of dopamine-producing neurons in a model relevant to Parkinson's disease therapy (McGuire et al., 2006). Moreover, blueberries and strawberries counter the deleterious effects of irradiation by reducing oxidative stress and inflammation, thereby improving neuronal signaling, preventing the accumulation of disease-related proteins such as tau in the hippocampus of irradiated rats (Poulose et al., 2014).

Indeed Andres-Lacueva et al. (2005) examined whether different classes of polyphenols could be found in brain areas associated with cognitive performance following blueberry (BB) supplementation. Thus, 19 months old F344 rats were fed a control or 2% BB diet for 8–10 weeks and tested in the Morris Water Maze (MWM), a measure of spatial learning and memory. Several anthocyanins were found in the cerebellum, cortex, hippocampus, or striatum of the BB supplemented rats, but not the controls. Correlational analyses revealed a relationship between MWM performance in BB rats and the total number of anthocyanin compounds found in the cortex, suggesting that these compounds may deliver their antioxidant and signaling modifying capabilities centrally.

To support and clarify the antioxidants effects of this compound recently Çoban et al. (2015) investigated the consequence of whole fresh BB treatment at different percentages on oxidative stress in age-related brain damage model. The study showed that BB treatments, especially BB at higher percentage reduced malondialdehyde and Protein C levels and acetylcholinesterase activity and elevated glutathione (GSH) levels and GSH-Px activity, diminishing apoptosis and ameliorating histopathological findings in the brain of rats treated with D-galactose (GAL). The authors concluded that BB partially prevented the shift toward an imbalanced pro-oxidative status and apoptosis together with histopathological amelioration by acting as an antioxidant (radical scavenger) itself in GAL-treated rats (Çoban et al., 2015).

It has been reported that long-term treatment with blueberry has also a neuroprotective effect in attenuating cerebral ischemia/reperfusion (I/R) injury. Zhou et al. (2015) showed that 24 h after I/R, pterostilbene (a major component of blueberry) dose-dependently improved neurological function, reduced brain infarct volume, and alleviated brain oedema. The most effective dose was 10 mg/kg; the therapeutic time window was within 1 h after I/R and treatment immediately after reperfusion showed the best protective effect. The protective effect was further confirmed by the results that post-ischemic treatment with pterostilbene (10 mg/kg) significantly improved motor function, alleviated BBB disruption, increased neurons survival and reduced cell apoptosis in cortical penumbra after cerebral I/R. The authors also found that pterostilbene (10 mg/kg) significantly reversed the increased content of malondialdehyde and the decreased activity of superoxide dismutase in the ipsilateral hemisphere, with decrease of the oxidative stress markers 4-hydroxynonenal and 8-hydroxyguanosine positive cells in the cortical penumbra. Then pterostilbene dose- and time-dependently exerts a neuroprotective effect against acute cerebral I/R injury (Zhou et al., 2015) and its antioxidant action is mediated by the increased expression of Nrf2 (Saw et al., 2014).

### Curcumin

Curcumin, the principal curcuminoid and the most active component in turmeric, is a biologically active phytochemical.

Several beneficial effects of curcumin for the nervous system have been reported. In an animal model of stroke curcumin treatment protected neurons against ischemic cell death and ameliorated behavioral deficits (Wang et al., 2005). A hormetic mechanism of action of curcumin is suggested from studies showing that levels of expression of the stress response protein HO-1 were increased in cultured hippocampal neurons treated with curcumin (Scapagnini et al., 2006). Moreover, curcumin has been shown to reverse chronic stress-induced impairment of hippocampal neurogenesis and increase expression of BDNF in an animal model of depression (Xu et al., 2007). At non-toxic concentrations, curcumin induces HO-1 expression by activating the Nrf2/ARE pathway both *in vitro* (Pae et al., 2007) and *in vivo* (Farombi et al., 2008).

Several studies also showed that curcumin interacts with NF-κB, and through this interaction exerts protective function also in the regulation of T-cell-mediated immunity (Kou et al., 2013). Recently González-Reyes et al. (2013) identified curcumin as a neuroprotector against hemin-induced damage in primary cultures of cerebellar granule neurons of rats. Hemin, the oxidized form of heme, is a highly reactive compound that induces cellular injury. Pre-treatment of the neurons with 5–30 μM curcumin increased by 2.3–4.9-fold HO-1 expression and by 5.6–14.3-fold GSH levels. Moreover, 15 μM curcumin attenuated by 55% the increase in ROS production, by 94% the reduction of GSH/glutathione disulfide ratio, and by 49% the cell death induced by hemin. Furthermore, it was found that curcumin was capable of Nrf2 translocation into the nucleus, suggesting that the pre-treatment with curcumin induces Nrf2 and an antioxidant response that may play an important role in the protective effect of this antioxidant against hemin-induced neuronal death (González-Reyes et al., 2013).

In rodents and human cells, curcumin-induced HO-1 overexpression was correlated with production of mitochondrial ROS, activation of transcription factors Nrf2 and NF-κB, induction of Mitogen-activated protein kinase (MAPK) p38 and inhibition of phosphatase activity (Andreadi et al., 2006; McNally et al., 2007). Moreover, curcumin is an activator of Nrf2 (Moi et al., 1994) by changing specific highly reactive cysteine residues of Keap1 (Dinkova-Kostova et al., 2002, 2005), with consequent lost ability of Keap1 to target Nrf2 for degradation, which then undergoes nuclear translocation. By using an Alzheimer transgenic mouse model (Tg2576), Lim et al. (2001) shown that dietary curcumin *in vitro*, inhibited aggregation as well as disaggregated fibrillary Amyloid beta (Aβ). *In vivo* studies showed that curcumin injected peripherally into aged mice crossed the BBB and directly bound small β-amyloid species to block aggregation and fibril formation *in vitro* and *in vivo*. These data suggest that low dose curcumin effectively disaggregates Aβ as well as prevents fibril and oligomer formation, supporting the rationale for curcumin use in clinical trials (Lim et al., 2001).

More recently, Garcia-Alloza et al. (2007) in transgenic APPswe/PS1dE9 mice demonstrated that curcumin, given intravenously for 7 days, crosses the BBB, binds to β-amyloid deposits in the brain and accelerates their rate of clearance (Garcia-Alloza et al., 2007).

Curcumin was also demonstrated to exert a neuroprotective effect in rats who underwent ischemia/reperfusion injury and this effect has been related to the direct scavenger effect of curcumin as well as to a curcumin-induced interference with the apoptotic machinery, increase in antioxidant molecules (GSH) and enzymes such as CAT and SOD (Al-Omar et al., 2006; Calabrese et al., 2008).

### Sulforaphane

Sulforaphane (SFN), a phytochemical present in high amounts in cruciferous vegetables such as broccoli, is known to activate the Nrf2-ARE stress response pathway in rodent brains and microvasculature and by this to reduce brain damages in a traumatic brain injury model (Zhao et al., 2007). Sulforaphane has been reported to protect cultured neurons against oxidative stress (Kraft et al., 2004), and dopaminergic neurons against mitochondrial toxins (Han et al., 2007; Son et al., 2008). This compound administration initiated at 1 h post-cortical impact injury has been shown to improve cognitive function, in particular spatial learning and memory, and to reduce working memory dysfunction (Dash et al., 2009).

In a model of neonatal hypoxia-ischemia, pretreatment with SFN increased the expression of Nrf2 and HO-1 in the mouse brain and reduced infarct ratio (Ping et al., 2010). Numerous other non-nutrients contained in food and plants have been ascribed to the list of Nrf2 activators, and among these several food-contained antioxidant polyphenols. One of the most important aspects of current polyphenol research is the focus on the neuroprotective capacity endowed by these molecules that seems to be due mostly to their ability to activate different defensive molecular pathways, instead to involve just their intrinsic antioxidant properties (Scapagnini et al., 2011).

In this regard, it has been recently demonstrated the critical role of Nrf2/HO-1 activation by some of these neuroprotective compounds, providing insight into the possible therapeutic significance of a closely related group of polyphenols against neurodegenerative disorders and cognitive decline (Scapagnini et al., 2011).

### Resveratrol

Neuroprotective effects of resveratrol have been reported by several different studies, in particular on beta-amyloid-induced oxidative cell death (Jang and Surh, 2003) and against several different insults on dopaminergic neurons of midbrain slice cultures (Okawara et al., 2007).

In particular in cultured rat pheochromocytoma (PC12) cells Resveratrol attenuated Aβ-induced cytotoxicity, apoptotic features, and intracellular ROS accumulation. Moreover, Aβ transiently induced activation of NF-κB was suppressed by resveratrol pretreatment (Jang and Surh, 2003) suggesting a key role of the NF-κB inflammatory pathway in the Aβ deposition and a possible therapeutic function of resveratrol in mediating neuroprotection.

Resveratrol protects cortical neurons from oxidative stress-induced injury (Zhuang et al., 2003), and suppress alcohol-induced cognitive deficits and neuronal apoptosis (Tiwari and Chopra, 2013). In addition, resveratrol has been found to reduce the production of IL-1 beta and TNF-alpha induced by LPS or Aβ in the microglia (Capiralla et al., 2012; Zhong et al., 2012). Further studies showed that the powerful neuroprotective effect of resveratrol has also been confirmed in neurodegenerative disorders, such as Parkinson's disease, Alzheimer's disease (Albani et al., 2009), and in traumatic brain injury (Ates et al., 2007; Zhang et al., 2015).

Prozorovski et al. (2008) found that the treatment of neural progenitor cells (NPCs) with resveratrol mimicked oxidizing conditions and increased differentiation of NPCs toward astrocytes through a mechanism that requires Sirt1 (Prozorovski et al., 2008). Indeed subtle alterations of the redox state, found in different brain pathologies, regulate the fate of mouse NPCs through SIRT1. Mild oxidation or direct activation of SIRT1 suppressed proliferation of NPCs and directed their differentiation toward the astroglial lineage at the expense of the neuronal lineage, whereas reducing conditions had the opposite effect. Under oxidative conditions *in vitro* and *in vivo*, Sirt1 was upregulated in NPCs, bound to the transcription factor Hes1 and subsequently inhibited pro-neuronal Mash1. In response to brain injury, NPCs differentiate preferentially into astrocytes rather than neurons. Excessive astrocyte expansion, known as astrogliosis, can prevent growth of neurons and interfere with proper damage repair. Therefore, the ability to direct differentiation of NPCs may be useful in protecting the brain against inflammatory diseases, such as multiple sclerosis, which involve astrogliosis (Prozorovski et al., 2008).

Indeed resveratrol was shown to affect the activity of SIRT1 *in vitro* on depending to the nature of the substrate for deacetylation (Baur and Sinclair, 2006). It has been reported that the SIRT1 agonist resveratrol protects *C. elegans* neurons expressing a fragment of the Huntington disease-associated protein huntingtin and mammalian neurons from mutant polyglutamine cytotoxicity in a HdhQ111 knock-in mouse model of Huntington disease (Dali-Youcef et al., 2007).

Moreover, resveratrol had no effect on the binding of NF-κB proteins to the DNA, but it blocked the TNF-induced translocation of p65 subunit of NF-κB and reporter gene transcription. Similarly, the activation of c-Jun N-terminal kinases (JNK) and its upstream MAPK are inhibited by resveratrol, which may explain the mechanism of suppression of AP-1 by resveratrol (Rahman et al., 2006).

Recently Zhang et al. (2015) investigated the potential role of resveratrol in attenuating hypoxia-induced neurotoxicity via its anti-inflammatory actions through *in vitro* models of the BV-2 microglial cell line and primary microglia. The authors found that resveratrol significantly inhibited hypoxia-induced microglial activation and reduced subsequent release of pro-inflammatory factors. In addition, resveratrol inhibited the hypoxia-induced degradation of I kappa B-alpha (IκB-alpha) and phosphorylation of p65 NF-κB protein. Importantly, treating primary cortical neurons with conditioned medium (CM) from hypoxia-stimulated microglia induced neuronal apoptosis, which was reversed by CM co-treated with resveratrol. Taken together, the results of this study suggest that resveratrol exerts neuroprotection against hypoxia-induced neurotoxicity through its anti-inflammatory effects in microglia. These effects were mediated, at least in part, by suppressing the activation of NF-κB, extracellular-signal-regulated kinases (ERK), and JNK/MAPK signaling pathways (Zhang et al., 2015).

Although several studies reported an efficacy of these compounds in animal model and *in vitro*, few plant-based products have been assessed in methodologically adequate human trials (Kennedy and Wightman, 2011), and clinical experiments have often failed to demonstrate any convincing therapeutic potency of these compounds (Berger et al., 2012).

## Dietary phytochemicals on cognitive performance in human studies

Accumulated data strongly suggest that phytochemicals from fruits, vegetables, herbs, and spices may exert relevant immunomodulatory and/or anti-inflammatory activities in the context of brain aging. The benefits of these substances for the cognitive health of older adults have been reported in several studies (Davinelli et al., 2015). In a recent review Shukitt-Hale (2012) highlighted the potential benefits of blueberries as a compound to impact age-related changes in neuronal aging. Additionally, Devore et al. (2012) shown that greater self-reported intakes of blueberries and strawberries were associated with slower rates of cognitive decline. Although several evidences point toward the beneficial effects of these substances, limitations of these researches include the use of correlational data as well as the lack of assessment of the bioavailability of these polyphenolic compounds from diets (Rowland et al., 2000).

Commenges et al. (2000) demonstrated that the intake of flavonoids in 1367 subjects over 65 years old was inversely associated with the risk of dementia at a 5-years follow-up. Recently Small et al. (2014) conducted a double-blind, placebo-controlled clinical trial using a pill-based nutraceutical (NT-020) that contained blueberry, carnosine, green tea, vitamin D3, and Biovin to evaluate the impact on changes in cognitive functioning. One hundred and five cognitively intact adults aged 65–85 years of age were randomized to receive NT-020 (*n* = 52) or a placebo (*n* = 53). Participants were tested with a battery of cognitive performance tests that were classified into six broad domains (episodic memory, processing speed, verbal ability, working memory, executive functioning, and complex speed) at baseline and 2 months later. The results indicated that persons taking NT-020 improved significantly on two measures of processing speed across the 2-month test period compared to persons on the placebo whose performance did not change. The authors concluded that the results were promising and suggest the potential for interventions like these to improve the cognitive health of older adults (Small et al., 2014).

Indeed these results have been confirmed by recent evidence by Rabassa et al. (2015). In the context of the Invecchiare in Chianti (InCHIANTI), a cohort study with 3 years of follow-up, the authors assessed the total urinary polyphenol (TUP) and the total dietary polyphenol (TDP) concentrations in 652 individuals without dementia aged 65 and older, and assessed cognition using the Mini-Mental State Examination (MMSE) and Trail-Making Test (TMT) at baseline and after 3 years of follow-up. Higher TUP levels were associated with lower risk of substantial cognitive decline on the MMSE and on the TMT-A, in a logistic regression model adjusted for baseline cognitive score and potential confounding factors. These findings showed that high concentrations of polyphenols were associated with lower risk of substantial cognitive decline in an older population studied over a 3-year period, suggesting a protective effect against cognitive impairment (Rabassa et al., 2015).

In a prospective study conducted among Japanese Americans living in the King County of Washington, Dai et al. (2006) found that frequent drinking of fruit and vegetable juices was associated with a substantially decreased risk of Alzheimer's disease, with an inverse association stronger after adjustments for potential confounding factors, and evident in all strata of selected variables. These findings suggest that fruit and vegetable juices may play an important role in delaying the onset of Alzheimer's disease (Dai et al., 2006).

Krikorian et al. (2010a,b) investigated the effects of daily consumption of wild blueberry juice in a sample of nine older adults with early memory changes. At 12 weeks, improved paired associate learning and word list recall were observed. In addition, there were trends suggesting reduced depressive symptoms and lower glucose levels. Instead, twelve older adults with memory decline but not dementia were enrolled in a randomized, placebo-controlled, double blind trial with Concord grape juice supplementation for 12 weeks (Butchart et al., 2011). The authors observed significant improvement in a measure of verbal learning and non-significant enhancement of verbal and spatial recall. There was no appreciable effect of the intervention on depressive symptoms and no effect on weight or waist circumference. Then these findings suggested that supplementation with Concord grape juice may enhance cognitive function for older adults with early memory decline.

In a more recent study (Devore et al., 2012), performed on 16,010 women aged ≥70 years, greater intakes of blueberries and strawberries were associated with slower rates of cognitive decline after adjusting for multiple potential confounders. Berry intake appeared to delay cognitive aging by up to 2.5 years. Additionally, in further supporting evidence, greater intakes of anthocyanidins and total flavonoids were associated with slower rates of cognitive decline in elder women (Devore et al., 2012).

On the other hand Butchart et al. (2011) investigated the same issue but with control for possible confounding factors as prior intelligence quotient (IQ). In a cross-sectional survey of 1091 men and women born in 1936, in which IQ was measured at age 11 years, at the age of 70 years, participants carried out various neuropsychological tests and completed a Food Frequency Questionnaire. Total fruit, citrus fruits, apple, and tea intakes were initially found to be associated with better scores in a variety of cognitive tests, but the associations were no longer statistically significant after adjusting for confounding factors, including childhood IQ, not supporting a role for flavonoids in the prevention of cognitive decline in later life (Butchart et al., 2011).

However, in all of these studies, no specific information on long-term dietary habits was available, while, long-term diet is likely to be most relevant for cognitive decline (Devore et al., 2012).

An epidemiological study (Ringman et al., 2012) suggested that curcumin, as one of the most prevalent nutritional and medicinal compounds used by the Indian population, is responsible for the reduced (4.4-fold) prevalence of AD in India compared to United States.

As seen above, although there are many experimental *in vitro* and *in vivo* evidence of the efficacy of curcumin in the prevention of neurodegeneration, at present very few human studies have been performed, which have shown some utility of this compound.

Ringman et al. (2012) performed a 24-week randomized, double blind, placebo-controlled study of curcumin with an open-label extension to 48 weeks. Thirty-six persons with mild-to-moderate AD were randomized to receive placebo, 2 g/day, or 4 g/day of oral curcumin for 24 weeks. For weeks 24 through 48, subjects that were receiving curcumin continued with the same dose, while subjects previously receiving placebo were randomized in a 1:1 ratio to 2 g/day or 4 g/day. At the end of the study no differences were found between treatment groups in clinical or biomarker efficacy measures (Ringman et al., 2012).

Indeed Cox et al. (2015) in a randomized, double blind, placebo-controlled trial examined the acute (1 and 3 h after a single dose), chronic (4 weeks), and acute-on-chronic (1 and 3 h after single dose following chronic treatment) effects of solid lipid curcumin formulation (400 mg) on cognitive function, mood and blood biomarkers in 60 healthy adults aged 60–85. One hour after administration curcumin significantly improved performance on sustained attention and working memory tasks, compared with placebo. Working memory and mood (general fatigue and change in state calmness, contentedness and fatigue induced by psychological stress) were significantly better following chronic treatment. A significant acute-on-chronic treatment effect on alertness and contentedness was also observed (Cox et al., 2015).

All together these results highlight the need for further investigation on the potential cognitive benefits of curcumin, especially in elderly, and the importance of the dose and method of administration.

Although the plant-derived polyphenol resveratrol has been shown to increase memory performance in primates, also for this compound interventional studies in older humans are lacking.

In a study by Kennedy et al. (2010) the effects of oral resveratrol on cognitive performance and localized cerebral blood flow variables in healthy human adults were assessed. In this very interesting randomized, double blind, placebo-controlled, crossover study, 22 healthy adults received placebo and 2 doses (250 and 500 mg) of trans-resveratrol in counterbalanced order on separate days. After a 45-min resting absorption period, the participants performed a selection of cognitive tasks that activate the frontal cortex for an additional 36 min. Cerebral blood flow and hemodynamic, as indexed by concentration changes in oxygenated and deoxygenated hemoglobin, were assessed in the frontal cortex throughout the post-treatment period with the use of near-infrared spectroscopy. The presence of resveratrol and its conjugates in plasma was confirmed by HPLC after the same doses in a separate cohort (*n* = 9). Resveratrol administration resulted in dose-dependent increases in cerebral blood flow during task performance. There was also an increase in deoxyhaemoglobin after both doses of resveratrol, which suggested enhanced oxygen extraction that became apparent toward the end of the 45-min absorption phase and was sustained throughout task performance. Cognitive function was not affected. Resveratrol metabolites were present in plasma throughout the cognitive task period, suggesting that single doses of orally administered resveratrol can modulate cerebral blood flow variables (Kennedy et al., 2010).

Witte et al. (2014) tested whether supplementation of resveratrol would enhance memory performance in older adults and addressed potential mechanisms underlying this effect. Twenty-three healthy overweight older individuals treated for 26 weeks with 200 mg/d resveratrol were compared to 23 participants that received placebo. Before and after the intervention/control period, subjects underwent memory tasks and neuroimaging to assess volume, microstructure, and functional connectivity (FC) of the hippocampus, a key region implicated in memory functions. In addition, anthropometry, glucose and lipid metabolism, inflammation, neurotrophic factors, and vascular parameters were assayed. The authors observed a significant effect of resveratrol on retention of words over 30 min compared with placebo. In addition, resveratrol led to significant increases in hippocampal FC, and the increases in FC between the left posterior hippocampus and the medial prefrontal cortex correlated with increases in retention scores. Then these finding could offer the basis for novel strategies to maintain brain health during aging (Witte et al., 2014).

## Conclusions

Despite the translational gap between basic and clinical research, the current understanding of the molecular interactions between phytochemicals, immune function, and inflammatory response could help in designing effective nutritional strategies to delay brain aging and improve cognitive function.

Although, as described, many studies demonstrate the efficacy *in vitro* and *in vivo* of phytochemicals (Table 1) in the prevention and treatment of cognitive disorders, even few evidence of their efficacy are available in humans, and especially still significant differences in the protocols used, dosages and in the different way of administration. Therefore, it seems that these results do not allow to finalize, which is the real efficacy of these compounds to prevent and to prevent and delay neuroinflammation associated with aging. Further research, mainly conducted with randomized controlled trials, should be performed in humans to determine the real role that phytochemicals can play in the prevention and treatment of neuroinflammaging.

**Table 1:** **Summary of phytochemicals with their food origin, effects in brain, studies demostrating this effects, the models used and their capability to cross the Blood-Brain Barrier (BBB)**.

**Phytochemicals**	**Food origin**	**Effects in neuroprotection**	**Methods**	**References**	**Blood-Brain Barrier passage**
Ferulic acid	Tomatoes	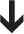 Prostaglandin E2 and TNF-α,	*in vitro* cromatography	Ou et al., 2003	Ferulic acid was transported across a model Blood-Brain Barrier (BBB). After administration of Shunaoxin pills, ferulic acid was rapidly absorbed and distributed in brain.
	Sweet corn, rice,	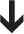 iNOS expression and function	Primary mesangial cell cultures	Tetsuka et al., 1996	
	Wheat oats, barley grain,	Inhibits microglial activation	Imprinting Control Region (ICR) strain mice	Kim et al., 2004	
	Chinese water chestnut, pineapple	Protects against changes in the conformation of synaptosomal membrane proteins	Cultured neuronal cells	Kanaski et al., 2002	Wu et al., 2014
	Seeds of coffee, apple	Protects against Aβ toxicity directly and by inducing protecting genes	Hippocampal cultures	Sultana et al., 2005	
	Artichoke, peanut	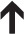 Hemeoxygenase-1 and heat shock protein 70	Rat astrocytes and neurons	Scapagnini et al., 2004	
	Orange, navy bean	Ameliorated neuroinflammation in including β-amyloid plaque-associated gliosis and expression of TNF-α and IL-1β and 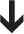 mRNA expression of superoxide dismutase 1, catalase, and GSH-Px 1	Mouse model of cerebral amyloidosis mutant human transgenes and *in vitro* mutant human APP-overexpressing murine N2a neuron-like cells.	Mori et al., 2013	
Epigallocatechin gallate (EGCG)	Black tea, green tea	Protects dopaminergic neurons from damage induced by 6-hydroxydopamine	Unilateral 6-hydroxydopamine (6-OHDA)-treated rat model of Parkinson's disease	Guo et al., 2007	The level of EGCG found in the major organs was found to be ~1/10 that found in the serum. Most interestingly, this includes the brain, suggesting that EGCG passes through the blood-brain barrier.
	Oolong teas, carob flour	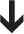 Mutant huntingtin misfolding and neurotoxicity	Transgenic flies and yeast cultures	Ehrnhoefer et al., 2006	
	Pecans, filberts, hazelnuts	Protects directly neurons against Aβ toxicity	Mixed (glial/neuronal) hippocampal cultured cells from E19 fetuses obtained from Sprague-Dawley rats	Bastianetto et al., 2000	Smith, 2011
	Raw cranberries, pistachios	Protects against Aβ-induced cognitive impairment	5-week-old male rats	Haque et al., 2008	
		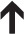 HO-1 expression by activation of the Nrf2-ARE pathway	Bovine aortic endothelial cells	Wu et al., 2006	
		Protects cultured rat cerebellar granule neurons from oxidative stress	Primary cultures of rat cerebellar granule neurons	Schroeder et al., 2009	
		Promotes cleavage of APP into α-CTF and soluble APP-α.	Primary micloglial and neurons cultures from Tg2576 mice	Obregon et al., 2006	
		Cleavage of APP into α-CTF and soluble APP-α with elevated α-secretase cleavage activity and activation of ADAM10	Mouse brains	Rezai-Zadeh et al., 2008	
Pterostilbene	Blueberry, eanuts, almonds	Reverses age-related deficits in neuronal and behavioral parameters	Male Fischer 344 rats	Joseph et al., 1999	It is generally assumed that Pterostilbene can cross the BBB due to its structural similarities to resveratrol.
	Grapes	Prevents learning and memory deficits	APP/PS1 transgenic mice	Joseph et al., 2003	
		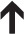 Survival of dopamine-producing neurons	Embryonic dopamin neurons transplanted into the unilaterally dopamin-depleted striatum	McGuire et al., 2006	Temsamani et al., 2015
		Improves neuronal signaling, preventing accumulation of proteins tau in the hippocampus of irradiated rats	Rats exposed to 1.5 Gy of 56Fe particles	Poulose et al., 2014	Andres-Lacueva et al., 2005
		Delivers antioxidant and signaling modifying capabilities centrally	19 months old F344 rats	Andres-Lacueva et al., 2005	
		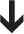 Malondialdehyde and Protein C levels, acetylcholinesterase activity and apoptosis 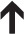 Glutathione (GSH) levels and GSH-Px activity	Rats treated with D-galactose	Çoban et al., 2015	
		Improved neurological function, 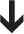 brain infarct volume, and oedema. 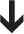 Malondialdehyde and 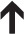 activity of superoxide dismutase in the ipsilateral hemisphere, with 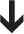 of 4-hydroxynonenal and 8-hydroxyguanosine	Male Kunming mice with induced focal cerebral ischemia	Zhou et al., 2015	
Curcumin	Curry, Worcestershire sauce	Protects neurons against ischemic cell death and ameliorated behavioral deficits	Mongolian gerbils	Wang et al., 2005	In transgenic APPswe/PS1dE9 mice demonstrated that curcumin, given intravenously for 7 days, crosses the BBB, binds to β-amyloid deposits in the brain and accelerates their rate of clearance.
	Food additive (E100)	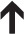 Expression of HO-1	Cultured hippocampal neurons	Scapagnini et al., 2006	
		Reverse chronic stress-induced impairment of hippocampal neurogenesis and 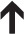 expression of brain-derived neurotrophic factor	Chronically stressed rats	Xu et al., 2007	
		Protective function in T-cell-mediated immunity	Male Sprague–Dawley rats	Kou et al., 2013	Garcia-Alloza et al., 2007
		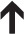 HO-1 expression and GSH levels	Primary cultures of cerebellar Granule neurons of rats	González-Reyes et al., 2013	
		Disaggregates Aβ as well as prevents fibril and oligomer formation	Alzheimer transgenic APPSw mouse model	Lim et al., 2001	
		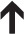 GSH and catalase, superoxide dismutase	Adult male Wistar albino rats, Male Sprague Dawley rats	Al-Omar et al., 2006; Calabrese et al., 2008	
Sulforaphane	Broccoli, brussels Sprouts	Activate Nrf2-ARE stress response pathway	Male Sprague Dawley rats and nrf2 –/– mice	Zhao et al., 2007	Various studies in animal models suggest the ability of Sulforaphane to cross the BBB and to accumulate in cerebral tissues.
	Cabbage cauliflower	Protects cultured neurons against oxidative stress	ARE–human placental alkaline phosphatase transgenic mice	Kraft et al., 2004	
	Kale, collard greens, horseradish	Protects dopaminergic neurons against mitochondrial toxins	CATH.a cells	Han et al., 2007	Jazwa et al., 2011
		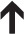 Spatial learning and memory, and 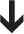 Working memory dysfunction	Male Sprague Dawley rats	Dash et al., 2009	Clarke et al., 2011
		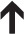 Nrf2 and HO-1 expression	Neonatal hypoxia-ischemia in Sprague–Dawley rat pups	Ping et al., 2010	
Resveratrol	Red Grapes, Peanut Butter, Dark Chocolate, Itadori Tea	Attenuated beta-amyloid-induced cytotoxicity, apoptotic features, and intracellular ROS accumulation. Beta-amyloid transiently induced activation of NF-κB was suppressed	PC12 cells	Jang and Surh, 2003	Acute administration of resveratrol by oral gavage using a low dose of 80 μg/kg results in significant accumulation in brain within 4 h. Short term treatment using a concentration of 40 μg/kg by the same route of administration for a period of 15 days also increases resveratrol content in the brain.
	Blueberries	Neuroprotection against dopaminergic neurons	Organotypic midbrain slice cultures	Okawara et al., 2007	
		Protects cortical neurons from oxidative stress-induced injury	Cultures of cortical neuronal cells isolated from embryos of timed pregnant mice	Zhuang et al., 2003	
		Suppress alcohol-induced cognitive deficits and neuronal apoptosis	Adult male Wistar rats	Tiwari and Chopra, 2013	
		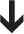 The production of IL-1 beta and TNF-alpha induced by LPS or Aβ in the microglia	The mouse microglial cell line BV-2	Capiralla et al., 2012; Zhong et al., 2012	Bertelli et al., 1999
		Resveratrol mimicked oxidizing conditions in neural progenitor cells	Mouse neural progenitor cells	Prozorovski et al., 2008	Resveratrol being a lipophilic compound can readily cross the BBB via transmembrane diffusion (Lin et al., 2010).
		Protects *C. elegans* neurons expressing a fragment of the Huntington disease-associated protein huntingtin and mammalian neurons from mutant polyglutamine cytotoxicity	HdhQ111 knock-in mouse model of Huntington disease	Dali-Youcef et al., 2007	Resveratrol, with its molecular weight of 228 Da (Amri et al., 2012) and lipid soluble properties, should easily cross the BBB.
		Inhibits hypoxia-induced degradation of I kappa B-alpha and phosphorylation of p65 NF-κB protein. These effects were mediated by suppressing the activation of NF-κB, extracellular-signal-regulated kinases (ERK) and JNK/MAPK signaling pathways	*In vitro* models of the BV-2 microglial cell line and primary microglia	Zhang et al., 2015	

## Author contributions

GC contributed substantially to conception, drafting the article, and final approval; VC contributed substantially to revision for important intellectual content and final approval of the version to be published; SD made substantial contributions to revising the article; GS made substantial contributions to revising the article; AF and NF contributed substantially to revision for important intellectual content and final approval of the version to be published. All the authors gave final approval of the version to be published.

### Conflict of interest statement

The authors declare that the research was conducted in the absence of any commercial or financial relationships that could be construed as a potential conflict of interest.
